# Time to eRAASe chronic inflammation: current advances and future perspectives on renin-angiotensin-aldosterone-system and chronic intestinal inflammation in dogs and humans

**DOI:** 10.3389/fvets.2023.1180125

**Published:** 2023-06-29

**Authors:** Romy M. Heilmann, Georg Csukovich, Iwan A. Burgener, Franziska Dengler

**Affiliations:** ^1^Department for Small Animals, College of Veterinary Medicine, University of Leipzig, Leipzig, Germany; ^2^Small Animal Internal Medicine, University of Veterinary Medicine Vienna, Vienna, Austria; ^3^Institute of Physiology, Pathophysiology and Biophysics, University of Veterinary Medicine Vienna, Vienna, Austria

**Keywords:** alternative RAAS, chronic inflammatory enteropathy, inflammatory bowel disease, electrolyte transport, enteroids, tight junctions

## Abstract

Chronic idiopathic intestinal inflammation is an increasing worldwide problem that affects companion animals, especially dogs, and human patients. Although these disease entities have been intensely investigated recently, many questions remain, and alternative therapeutic options are needed. Diarrhea caused by dysregulation of intestinal electrolyte transport and subsequent fluid and electrolyte losses often leads to secondary consequences for the patient. Currently, it is not exactly clear which mechanisms are involved in the dysregulation of intestinal fluid absorption, but differences in intestinal electrolyte shifts between human and canine patients suggest species-specific regulatory or counterregulatory mechanisms. Several intestinal electrolyte transporters are differentially expressed in human patients with inflammatory bowel disease (IBD), whereas there are virtually no studies on electrolyte transporters and their endocrine regulation in canine chronic inflammatory enteropathy. An important mechanism involved in regulating fluid and electrolyte homeostasis is the renin-angiotensin-aldosterone-system (RAAS), which may affect intestinal Na^+^ transport. While RAAS has previously been considered a systemic regulator of blood pressure, additional complex roles of RAAS in inflammatory processes have been unraveled. These alternative RAAS pathways may pose attractive therapeutic targets to address diarrhea and, thus, electrolyte shifts in human IBD and canine chronic inflammatory enteropathy. This article comparatively summarizes the current knowledge about electrolyte transport in human IBD and canine chronic inflammatory enteropathy and the role of RAAS and offers perspectives for novel therapeutic avenues.

## 1. Chronic intestinal inflammation—a one-health perspective

Human IBD—comprising mainly Crohn’s disease (CD) and ulcerative colitis (UC)—has a high prevalence in industrialized countries, and patients often experience severe distress and significantly reduced quality of life. Healthcare costs to treat IBD in humans are immense, amounting to 15–30 billion US dollars annually in the United States and about 5 billion Euros in Europe (1). The exact prevalence of chronic inflammatory enteropathy (CIE) in dogs is currently unknown, but it is estimated at 1%–2% in referral settings (2). CIE in dogs can range in severity and is subcategorized based on the response to treatment (2). In contrast to canine CIE, different compartments of the intestines are predominantly affected in patients with CD and UC, likely reflecting differences in the disease pathogenesis. Overt inflammatory responses are a common characteristic, resulting from environmental factors (dietary and microbial antigens) combined with a genetic predisposition (3). Dogs have accompanied humans and shared the human lifestyle for over 1,000 years, and it is thus not surprising that they develop similar civilization diseases. The prevalence of idiopathic IBD—either responsive (immunosuppressant-responsive enteropathy, IRE) or not responsive (non-responsive enteropathy, NRE) to immunosuppressive treatment—as a form of chronic inflammatory enteropathies (CIE) in dogs increased simultaneously with the rise of IBD in humans and both diseases share many characteristics, including pathogenesis and clinical signs (4–7). In dogs, CIE is characterized by chronic gastrointestinal signs, exclusion of other underlying diseases, and confirmation of gastrointestinal inflammation together with a response to treatment with either an elimination diet alone (food-responsive enteropathy, FRE) or in combination with immunosuppressant medication (IRE or NRE) (2, 6, 7). The resulting diarrhea and accompanying shifts in plasma electrolytes can severely compromise the dogs’ and their owners’ quality of life.

A hallmark of IBD is diarrhea due to intestinal hypersecretion and hampered reabsorption of electrolytes and fluid, often accompanied by serum electrolyte changes. Although the clinical signs are similar and largely overlapping, reports suggest different compensatory mechanisms to be activated both in the intestinal epithelium and on the systemic level in affected humans and dogs (8–10), which might also call for different therapeutic approaches. While hyponatremia is the most common electrolyte change in human IBD (11), hypokalemia appears more prevalent in canine CIE (9), suggesting species-specific compensatory mechanisms. A better understanding of the pathophysiologic mechanisms in dogs with CIE is expected to help identify novel therapeutic targets that could ameliorate diarrhea in affected dogs and be valuable for treating human IBD patients. While IBD in people has been under investigation for decades, significantly less is currently known about the pathophysiology of chronic idiopathic intestinal inflammation (CIE) in dogs.

## 2. Pathophysiology of diarrhea—gastrointestinal electrolyte transport and barrier formation

Central functions of the intestinal epithelium are the formation of a tight barrier to shield the host from luminal microbiota and other noxae and the vectorial transport of nutrients, electrolytes, and water. Uptake and secretion of nutrients and electrolytes are the major driving force for the (mostly paracellular) absorption and secretion of water. The gastrointestinal tract faces large fluid and electrolyte shifts, and the healthy intestinal mucosa absorbs about 98% of that fluid (12, 13). The (passive) movement of water is driven by the (active) uptake or secretion of electrolytes, primarily Cl^−^ and Na^+^. Due to its high absorptive capacity, the colonic epithelium can compensate for an increased secretion and/or defective absorptive capacity in the small intestine (14). Diarrhea develops if the compensatory capacity of the colon is exceeded and is often accompanied by serum electrolyte changes. The highest fecal water output is thus seen with disease involving the colon (12). Not surprisingly, diarrhea is invariably seen in humans with IBD, particularly in UC (15). In dogs, the lesions are typically more heterogeneously distributed in the gastrointestinal tract, and about 80% of affected animals show diarrhea (9). This lower prevalence of diarrhea [80% in dogs vs. 100% in people (9, 15)] might indicate a slightly more efficient compensation of intestinal malabsorption in dogs than in people.

Both increased secretion and reduced absorption of electrolytes cause diarrhea in human IBD patients (16). However, colonic absorption could still compensate for this if the colonic absorptive and re-absorptive transport mechanisms remain intact (17, 18). The main mechanisms for the uptake of luminal electrolytes—and thus the absorption of water—in the mammalian intestine is Na^+^-coupled cotransporters, particularly the Na^+^/H^+^-exchanger family (NHE) and the epithelial Na^+^ channel (ENaC). Both are downregulated in human IBD (19, 20) and rodent models of dextran-sulfate-sodium-induced colitis, along with the Na^+^/K^+^-ATPase that generates the gradient for the effective uptake of Na^+^ from the intestinal lumen (11, 20, 21), causing a decreased (re-)absorption of water. A knockout of NHE3, but not of NHE2, leads to diarrhea in a mouse model (22), and NHE3 was demonstrated to be the major isoform for Na^+^ absorption across the canine ileum epithelium (23).

This finding is especially interesting in conjunction with reports of increased serum aldosterone levels in human IBD patients (11, 24), suggesting a systemic attempt at a counter-regulation mediated by the renin-angiotensin-aldosterone system (RAAS) as ENaC, NHE3 and Na^+^/K^+^-ATPase are upregulated by aldosterone (25–27). Other transport proteins might also be involved in the dysregulation of intestinal fluid absorption, such as the anion exchangers putative anion transporter 1 (PAT1), down-regulated in adenoma (DRA), the Cl^−^ channel cystic fibrosis transmembrane conductance regulator (CFTR) (16, 28), monocarboxylate transporter 1 (MCT1) (11, 21), and anion exchanger 2 (AE2). The Na^+^/K^+^/2Cl^−^ cotransporter (NKCC) on the basolateral side of the epithelium might have a pivotal role in regulating the driving force for intestinal secretion [e.g., by CFTR and chloride channel 2 (CLC2)]. Similarly, basolateral K^+^ channels might be important in driving colonic secretion. The K^+^ channel KCNN4 is specifically upregulated in human IBD patients (29), and additional K^+^ channels or pumps may be located in the intestinal epithelial brush border membrane (16), but their role in human IBD (and canine CIE) is poorly understood. The effect of CIE on intestinal electrolyte transport in dogs has not been investigated to date.

Following established electrolyte gradients, the secretion and reabsorption of water mainly take the paracellular route. Therefore, the epithelial barrier formed by tight junction proteins is an important factor in the pathogenesis of diarrhea. Tight junctions and other cell–cell contacts are essential components located between adjacent epithelial and endothelial cells throughout the mammalian organism. In human IBD, the barrier-forming claudins 3, 4, 5, 7, and 8 are downregulated and disoriented from the plasma membrane, as are occludin and ZO-1, whereas the pore-forming claudin 2 is upregulated (30) along with increased paracellular permeability (20). In dogs with CIE, the expression of claudins or occludin is not altered in the duodenum, but colonic occludin mRNA levels are decreased (31). Apart from these findings, the regulation of tight junction proteins has yet to be investigated in dogs with CIE (32), but a thorough understanding of their role would be a major premise for further studying the pathomechanisms of CIE-related diarrhea in dogs. The colonic expression of occludin and claudin 8 is regulated (along with ENaC) by aldosterone (33), which may imply an additional therapeutic potential for RAAS in IBD and potentially also CIE in dogs.

## 3. Classical and alternative RAAS pathways—great complexity and far-reaching effects

RAAS has been extensively studied in cardiovascular and renal pathophysiology, and it appears to have much greater non-linear complexity than previously known (34). It acts on intestinal transport and barrier function, as described above. In addition, RAAS is involved in other intestinal functions, including the absorption of glucose and peptides, gastrointestinal motility, and the regulation of mesenteric blood flow (35, 36). Given the differences in electrolyte imbalances between canine CIE and human IBD patients, RAAS pathways might be differentially activated in these conditions.

**Figure 1:**
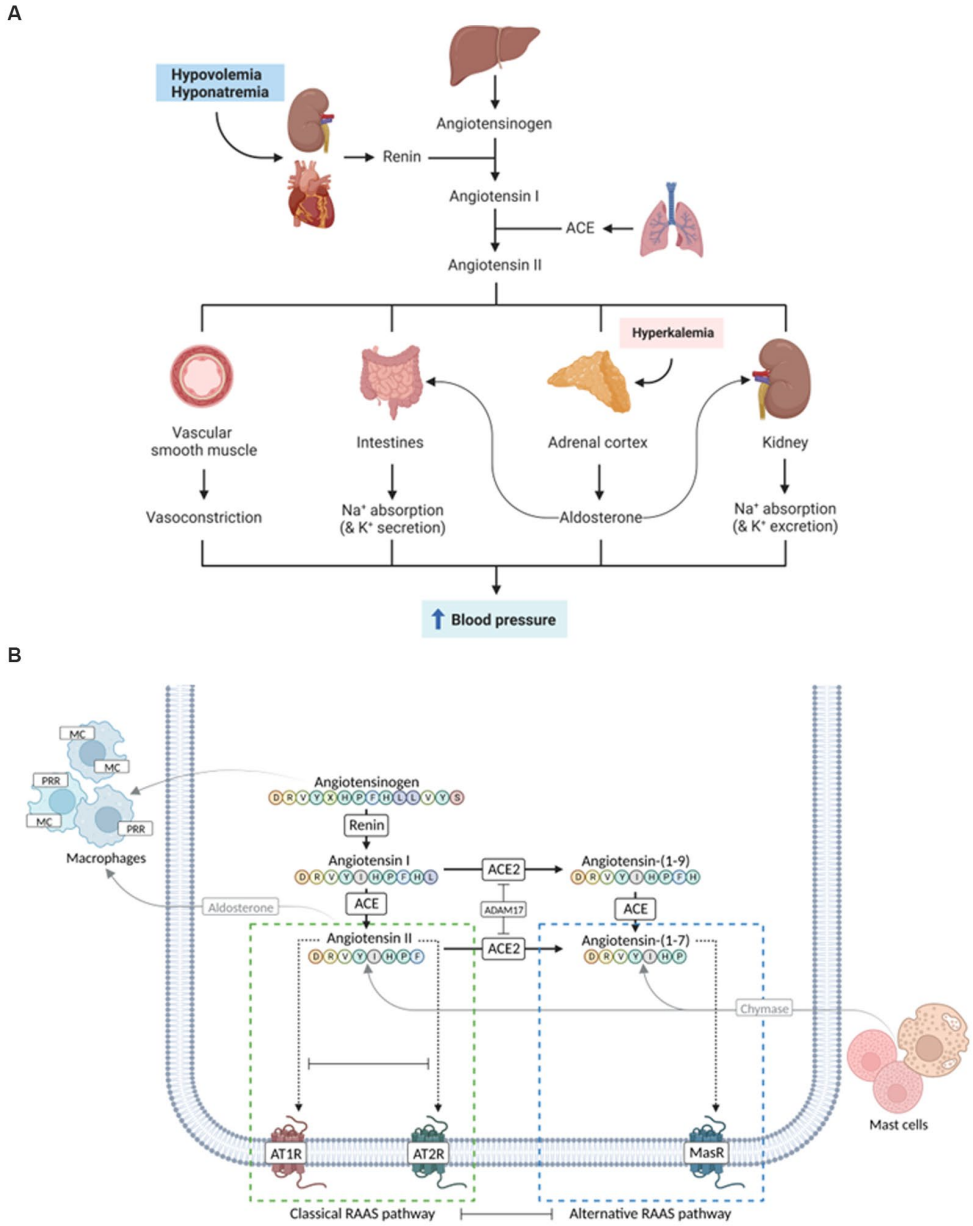
Evolution of the complexity of the renin-angiotensin-aldosterone system (RAAS). **(A)** Traditional simple view of the RAAS involving mostly cardiovascular and renal effects. **(B)** More recent complex view on classical and alternative RAAS pathways that might play a role in human inflammatory bowel disease (IBD) and/or canine chronic inflammatory enteropathy (CIE). Whereas the activation of the classical arm leads to vasoconstrictive, proinflammatory, profibrotic, and prothrombotic effects (green dashed box), components of the alternative RAAS pathways result in vasodilatory, anti-inflammatory, antifibrotic, and antithrombotic responses (blue dashed box). ACE, angiotensin-converting enzyme; ATR, angiotensin receptor; MR, mineralocorticoid receptor; PRR, prorenin receptor. Images created with BioRender.com.

Classically, renin cleaves angiotensinogen to angiotensin I (Ang I), which is then processed by angiotensin-converting enzyme (ACE) to the vasoconstrictor Ang II that activates aldosterone. This “traditional RAAS” has been well characterized as a circulatory blood pressure regulator (Figure 1A) and has presented a pharmacotherapeutic target for decades. In contrast, the existence of additional peptides derived from Ang I and II that constitute the “alternative RAAS” and their role in cardiovascular physiology and disease pathogenesis has long been neglected. The involvement of these recently discovered factors (Figure 1B) challenges the former simple concept of RAAS but also lends itself to potential novel therapeutic avenues beyond managing cardiovascular pathologies. Recent evidence also supports the coexistence of localized “tissue RAAS” mediating local (paracrine) effects.

Renin, a peptidase, represents the rate-limiting step in the RAAS cascade. After release from epithelioid cells of the renal juxtaglomerular apparatus into the circulation, renin cleaves an N-terminal decapeptide from angiotensinogen, a glycoprotein of the globulin superfamily synthesized in the liver and (though controversial) adipose tissue (37, 38), resulting in Ang I. The biologically active octapeptide Ang II results from the cleavage of Ang I by ACE, which is expressed primarily by pulmonary and renal endothelial cells and has also been detected in other tissues, including the myocardium and intestines (39). ACE is most active when bound to cell membranes. Together with the short half-life of Ang I and II, this indicates localized actions of RAAS (40). Similarly, an effect of renin and/or Ang II at the tissue level, rather than in the circulation, is supported by detecting (pro-)renin receptors in several tissues, such as the heart, brain, placenta, kidney, and liver (41).

The main effect of Ang II is an increase in systemic blood pressure by regulating vasoconstriction and cardiac output (42). As an intermediate effect, increased Na^+^ reabsorption in the proximal renal tubules (via NHE3) and induction of thirst and salt appetite, subsequently increasing extracellular volume and, thus, blood pressure, are induced (43–45). As a longer-term effect, Ang II stimulates (a) the expression and secretion of aldosterone, thus increasing the reabsorption of Na^+^ in the renal collecting ducts via ENaC on the gene expression level and (b) hypothalamic antidiuretic hormone (ADH, vasopressin) secretion leading to the insertion of aquaporins in the renal collecting ducts (25). Together, these mechanisms increase water reabsorption and thus blood volume and systemic blood pressure (Figure 1). It is important to recognize, however, that the enhanced reabsorption of Na^+^ in the collecting ducts causes a concurrent loss of K^+^ due to the extrusion of K^+^ via apical channels into the lumen of the renal collecting ducts, which is driven by the electrochemical gradient that increases with the reabsorption of Na^+^ (46).

Beyond these direct and indirect effects on systemic blood pressure, Ang II also elicits immunomodulatory effects by inducing proinflammatory cytokines and chemokines (e.g., TNFα, IL-6, and TGF-β1) in renal tubular cells and cells of the immune system (47–49). Ang II is also involved in hypertrophic remodeling (e.g., of the myocardium) by inducing cell proliferation and growth, but a direct effect of Ang II on extracellular matrix synthesis has also been observed (24, 47, 50). Thus, Ang II is presumed to be involved in the pathologic process of fibrogenesis (e.g., cardiac, renal, and hepatic fibrosis) (51, 52), which is also a major factor in the pathogenesis of human IBD (53). The binding of prorenin to its tissue receptor further contributes to myocardial fibrosis via the activation of intracellular signaling pathways (54, 55).

Four angiotensin-receptor (ATR) isoforms have been described, AT1R–AT4R. The ATRs are G-protein coupled transmembrane receptors (40) that might dictate the effects of Ang II by spatial differences in tissue abundance. AT1R is the primary receptor mediating the effects of Ang II and is expressed in most tissues, particularly the liver, adipose tissue, and placenta (39, 56). While AT1R is well characterized, the exact functions of the remaining three isoforms of ATR remain currently unknown. AT2R is found primarily during fetal development but may be upregulated under pathological conditions in adulthood (43), especially those affecting the lungs or smooth muscle (56). A vasodilatory effect of AT2R (i.e., opposing AT1R-mediated effects) has also been reported (40, 57, 58) and may provide a “safety net” preventing exaggerated and counterproductive effects of Ang II via AT1R.

Besides these traditional RAAS components, additional enzymes are described to act on Ang I and Ang II, representing the “alternative RAAS” (Figure 1B). To date, the best characterized is ACE2, which can cleave a nonapeptide, Ang (1–9), from Ang I or a heptapeptide, Ang (1–7), from Ang II (59, 60). Interestingly, one of the first observations of an alternative route of Ang I breakdown to Ang (1–7), independent from ACE, was in dogs (61). Ang (1–9) can also be converted to Ang (1–7) by ACE. Ang (1–7) responses can counteract those of Ang II [i.e., vasorelaxant, anti-proliferative, anti-inflammatory, anti-fibrotic, and thus likely (cardio-)protective] (59, 62, 63), presumably via binding to AT2R (54, 59). In hypertensive rats, Ang (1–7) reduced the heart rate but not systemic blood pressure (63). Simultaneously, the formation of Ang (1–7) from Ang II is inherent in decreased Ang II concentrations. With the discovery of Mas, an additional RAAS receptor was identified that might act as the main receptor for Ang (1–7) and thus the “alternative arm” of RAAS (59, 62, 64). The pathophysiologic role and effects of Ang (1–7) have raised hopes for a therapeutic application to address the adverse effects of Ang II in various pathologies. However, the pathways and effects of Ang II are currently still controversial and remain first to be clarified (63). Formation of Ang (2–8) (also referred to as Ang III) and Ang (3–8) (also known as Ang IV) has also been described (40). These peptides bind to AT1R and elicit similar effects as Ang II (54).

## 4. RAAS crossroads between adaptation, disease, and novel therapeutic targets

Components of the RAAS have paracrine and/or autocrine cytokine-like effects and regulate inflammation, tissue repair, and fibrosis (21, 65, 66), all important factors in the pathogenesis of canine CIE and human IBD. In addition to upregulating adhesion molecules, Ang II is chemotactic for inflammatory cells, particularly of the mononuclear lineage. These cells produce RAAS components following activation (mediated by IL-1, TNF-α, NF-κB, and/or PPARγ), resulting in a positive-feedback loop with the potential to perpetuate chronic inflammatory responses (66–68). Ang II also has profibrotic effects via TGF-β, connective tissue growth factor stimulation, and inhibition of matrix metalloproteinase (MMP)-mediated extracellular matrix degradation (69). While conflicting data exist on TGF-β expression in canine CIE depending on the gastrointestinal segment affected (4, 70,73), and unlike in humans stricturing behavior is not observed in affected dogs, intestinal mucosal MMP-2 and -9 activities are increased in canine CIE (74). Toll-like receptor (TLR) and RAGE (receptor for advanced glycation end products) expression are dysregulated in canine CIE (5, 75, 76), and RAAS blockade has anti-inflammatory effects by suppression of TLR2 and TLR4 in humans (77).

Inhibition of RAAS pathways [e.g., Ang II production by ACE inhibitors (ACEIs) or its effects by ATR blockers (ARBs)] could downregulate inflammatory mediators and the innate immune receptors TLR2, TLR4, and RAGE. This concept presents a novel therapeutic strategy that targets the inflammatory response in canine CIE and warrants further study. Classical and alternative RAAS pathways (Figure 1B) are complementary systems with the potential to oppose or compensate for the actions of the contralateral arm (60, 77, 78), and their balance (or imbalance) might play an important role in the pathogenesis of intestinal inflammation. Thus, a (receptor) specific approach is most promising for therapeutically targeting the RAAS. The alternative RAAS has anti-inflammatory properties (59, 60). Ang (1–7) is a promising therapeutic target that attenuated intestinal inflammation in a rodent model of IBD (78). Components of classical and alternative RAAS are expressed in the intestinal mucosa in humans (34, 78, 79), with disparate ACE2 imbalances in the small intestine (downregulation) and colon (upregulation) in IBD patients (59, 62, 79). ACE2, as the main enzyme for cleavage of Ang II to Ang (1–7) which neutralizes the pro-inflammatory and pro-fibrotic effects of Ang II, might be critical for mounting pro- vs. anti-inflammatory responses (80). It is expressed in the gastrointestinal tract in cats (81) but has not been investigated in dogs. Circulating ACE and ACE2 act as decoy receptors, and the plasma ACE2/ACE ratio is increased in people with IBD. Cleavage of ACE2 is controlled by the metalloprotease ADAM17 (34), and ACE2 induction by cardiovascular pathology—shifting the balance between Ang peptides in plasma—is more pronounced in dogs than people (82). MasR is expressed in the canine ileum (83) but remains to be investigated in canine CIE. Likewise, tissue prorenin receptor (PRR) and mineralocorticoid receptor (MR) expression (e.g., by macrophages), as well as chymase activation (e.g., by mast cells), can modulate local RAAS effects (Figure 1B) and inflammatory responses (43) but remain to be studied in canine CIE. ACEIs (decreasing the production of Ang II), Ang II blockade (antagonizing AT1R signaling), MR or PRR antagonists, and/or chymase inhibitors could be useful and inexpensive alternative or adjunct therapeutic options for chronic intestinal inflammation (39, 84, 85) and potentially other autoinflammatory diseases (e.g., autoimmune hepatitis) in dogs.

## 5. Discussion and conclusions

**Figure 2:**
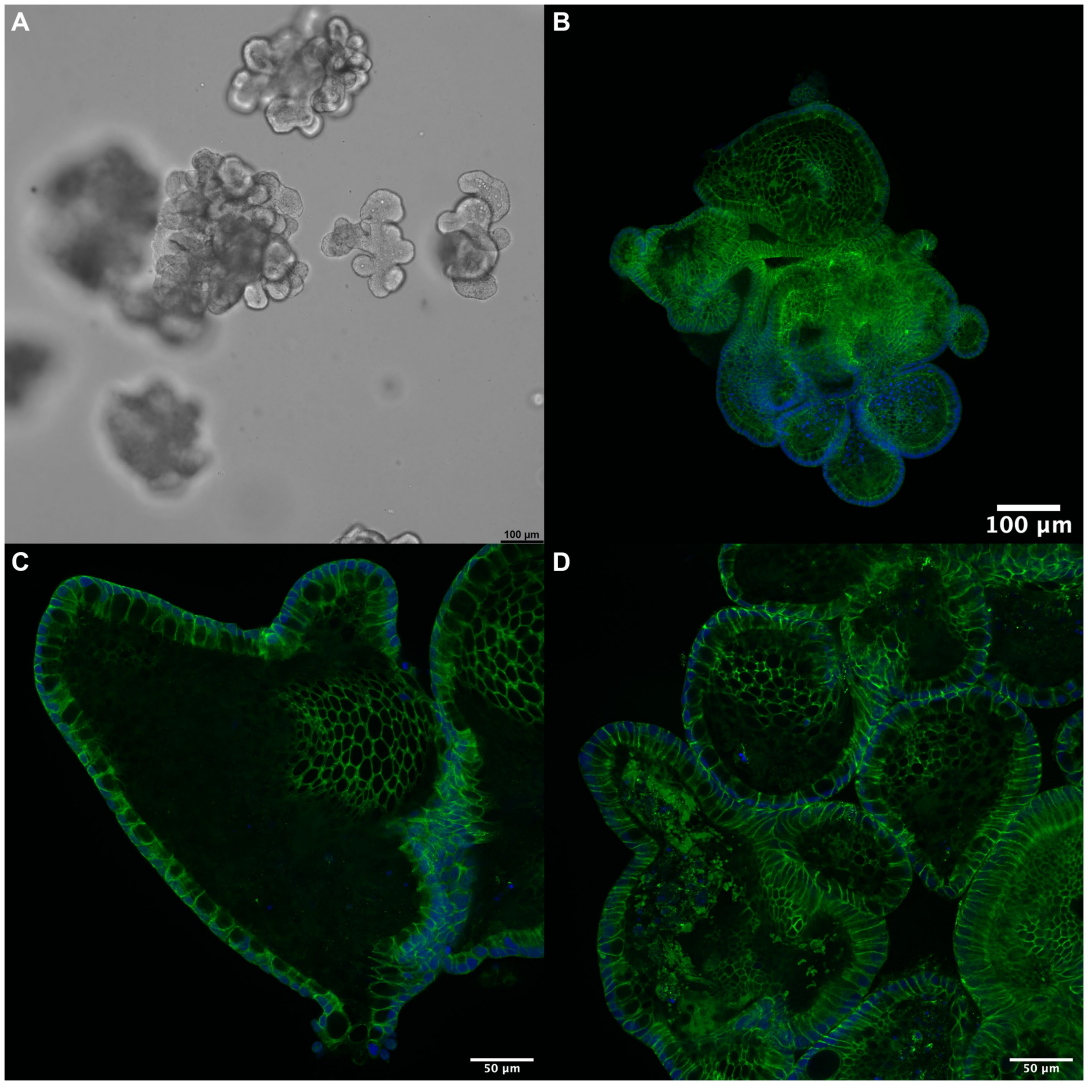
3D intestinal organoids for drug discovery. **(A)** Canine enteroids (shown in culture, phase contrast microscopy) pose an advanced *in vitro* model to investigate the pathophysiology of canine CIE further. These organoids allow the study of epithelial transport, inflammation, and barrier function: immunofluorescent staining (green) for **(B)** occludin, **(C)** claudin-1, and **(D)** claudin-7 indicates the formation of a functional polarized epithelium expressing tight junction proteins. Cell nuclei are counterstained with DAPI (blue). Scale bars: 100 μm **(A,B)** and 50 μm **(C,D)**.

Humans and dogs are close companions and share several civilization diseases, including idiopathic IBD and CIE. Although the shared Western lifestyle is proposed as a common denominator in the etiology of both conditions, there appear to be some species-specific differences in the disease characteristics, including the primary disease localization and distribution, resulting electrolyte changes, and potentially corresponding (counter-)regulatory mechanisms. While the current body of knowledge and research is more extensive for human IBD than canine CIE, a complete understanding of the underlying pathophysiology and possible mechanistic approach to therapy needs to be improved in both species. Exploration of alternative treatment options for dogs with CIE is needed as currently available drugs—particularly corticosteroids—carry significant side effects and biologicals (e.g., monoclonal antibodies against receptors or inflammatory cytokines) are not currently available (and very unlikely available soon) as a treatment option for canine CIE (86). Understanding commonalities and species-specific differences can be expected to result in the development of improved treatment strategies, and targeting RAAS might be one of these options. A thorough understanding of the role of RAAS pathways in the pathophysiology of canine CIE is needed to assess the therapeutic potential and potential side effects. Novel research methods, particularly canine intestinal organoids (Figure 2) that provide a reproducible and stable *in vitro* system for disease modeling and drug development (87–90), will be vital to further evaluate the effects of RAAS modifiers on epithelial ion transport, inflammatory responses, and intestinal barrier function comparatively. Organoids will allow to implement the 3R principles (6) and pave the way for urgently needed novel disease-specific treatment strategies in canine CIE and human IBD.

## Data availability statement

The original contributions presented in the study are included in the article/supplementary material, further inquiries can be directed to the corresponding author.

## Author contributions

RH, IB, and FD: conceptualization. RH and FD: manuscript draft. RH and GC: figures. All authors contributed to the article and approved the submitted version.

## Funding

This work was funded by a grant from the Leipzig veterinary junior scientist support program financed by the “Freundeskreis Tiermedizin,” the Faculty of Veterinary Medicine, and by Ceva Santé Animale. GC was funded by the Austrian Academy of Sciences (ÖAW), DOC fellowship grant number 26349.

## Acknowledgments

The immunofluorescent imaging was performed using resources of the VetCore Facility (VetImaging) at Vetmeduni, Austria. The authors also acknowledge support from the German Research Foundation (DFG) and the University of Leipzig within the program of Open Access Publishing.

## Conflict of interest

The authors declare that the research was conducted in the absence of any commercial or financial relationships that could be construed as a potential conflict of interest.

## Publisher’s note

All claims expressed in this article are solely those of the authors and do not necessarily represent those of their affiliated organizations, or those of the publisher, the editors and the reviewers. Any product that may be evaluated in this article, or claim that may be made by its manufacturer, is not guaranteed or endorsed by the publisher.
